# Mathematical modelling for variations of inbreeding populations fitness with single and polygenic traits

**DOI:** 10.1186/s12864-017-3492-1

**Published:** 2017-03-14

**Authors:** Shuhao Sun, Fima Klebaner, Tianhai Tian

**Affiliations:** School of Mathematical Sciences, Monash University, Melbourne, VIC 3800 Australia

## Abstract

**Background:**

Inbreeding mating has been widely accepted as the key mechanism to enhance homozygosity which normally will decrease the fitness of the population. Although this result has been validated by a large amount of biological data from the natural populations, a mathematical proof of these experimental discoveries is still not complete. A related question is whether we can extend the well-established result regarding the mean fitness from a randomly mating population to inbreeding populations. A confirmative answer may provide insights into the frequent occurrence of self-fertilization populations.

**Results:**

This work presents a theoretic proof of the result that, for a large inbreeding population with directional relative genotype fitness, the mean fitness of population increases monotonically. However, it cannot be extended to the case with over-dominant genotype fitness. In addition, by employing multiplicative intersection hypothesis, we prove that inbreeding mating does decrease the mean fitness of polygenic population in general, but does not decrease the mean fitness with mixed dominant-recessive genotypes. We also prove a novel result that inbreeding depression depends on not only the mating pattern but also genetic structure of population.

**Conclusions:**

For natural inbreeding populations without serious inbreeding depression, our theoretical analysis suggests the majority of its genotypes should be additive or dominant-recessive genotypes. This result gives a reason to explain why many hermaphroditism populations do not show severe inbreeding depression. In addition, the calculated purging rate shows that inbreeding mating purges the deleterious mutants more efficiently than randomly mating does.

## Background

In genetic terminology, inbreeding is the breeding of two individuals who are related to each other. Here inbred is defined as the individual that is generated from inbreeding. The closeness of individuals is important in inbreeding, which has substantial influence on the fitness of offspring individuals. Inbreeding depression refers to the reduced survival and fertility of offspring of related individuals. An important mechanism that leads to inbreeding depression is overdomain in which the heterozygote has greater phenotype value and perhaps is more fit than the homozygous state for either of the alleles that it comprises. Therefore there are two major approaches to increase homozygosity but decrease fitness. The first method is to increase homozygosity for partially recessive detrimental mutations; while the second approach is to increase homozygosity for alleles at loci with heterozygote advantage (overdominance). However, it is still under debate which mechanism is more dominat in nature [1–5]. This is an important question in genetics because two differnet mechanisms will lead to different theories regarding the trait values of the crossbred progeny. If the overdominance theory is valid, the mean trait value of crossed lines will return to the equivalence of the outbred population, as heterozygosity will be restored. On the other hand, the partial dominance theory predicts that the mean trait value will exceed that of the outbred population. In this case, in addition to the restore of heterozygosity, crossbred individuals will be purged from their genetic load [6, 7].

Genetic purging is the process for frequency reduction of a deleterious allele. It has been accepted that purging was achieved when inbreeding depression is caused by deleterious recessive alleles. A fitness rebound in inbred populations provides evidence to support the partial dominance mechanism [8, 9]. The effect of genetic purging have been observed in a number of experimental studies [10, 11], but overall, the evidence for purging in plant and animal populations is still limited. In addition, the combined effect of both inbreeding and purging is important for evaluating the evolutionary consequence of inbreeding and for making recommendations in conservation [12]. Although the inbreeding-purging model has been proposed to predict the evolution of the fitness and inbreeding load [13], a number of model parameters should be estimated from observation data. Thus it is high demanded to explore the role of purging in restoring fitness and calculation of purging speed.

The number of phenotypes produced for a given trait depends on how many genes control the trait. There are two types of trait. The single gene trait is controlled by a single gene that has two alleles. On the other hand, polygenic trait is controlled by two or more genes and has 2 or more alleles. Unlike monogenic traits, polygenic traits do not follow patterns of Mendelian inheritance (discrete categories). Instead, their phonotypes vary along a continuous gradient, such as the height of animals and the colour of human skin. Although we have conducted theoretical study for the single trait [14], the study of inbreeding depression for polygenic traits is more challenging and there is limited theoretical result regarding the variation of mean fitness under inbreeding.

In this work we will conduct novel theoretical studies for inbreeding depression in large population with Mendelian and polygenic traits. First, for single locus, we will explore the conditions for the monotonical increase of the mean fitness. We also derive a formula to calculate the purging rate. In addition, for multiple loci, we will find conditions for inbreeding mating reducing the mean fitness of the population. Furthermore, this work will show mechanisms to determine inbreeding depression. As an application, we present additional reasons for the frequent occurrence of self-fertilization populations. The well-known reason is that self-pollination does not need to be visited by animals to produce seed and hence have ecological advantageous under some circumstances [15].

## Methods

### Inbreeding of Mendelian traits

A Mendelian trait is a single trait that is regulated by a single locus and shows a simple discrete inheritance pattern. According to Wright’s formula [5, 16], at one autosomal locus of two alleles *A* and *a* with frequency *x* and 1−*x*, the three diploid genotypes *AA,Aa* and *aa* have frequency

$${} x^{2}+fx(1-x), \quad 2x(1-x)-2fx(1-x), \quad (1-x)^{2}+fx(1-x) $$

respectively, where *f* is the inbreeding coefficient that is defined as the probability that two homologous alleles in an individual are identical by descent (IBD) [5, 17]. In addition, the fitness of *AA,Aa* and *aa* is denoted as *w*_11_,*w*_12_,*w*_22_, respectively. Thus the change of frequency *Δ* *x*=*x* ^′^−*x* is

$$\begin{aligned} \Delta x &= \frac{x(1-x)}{\bar w}\left[\left(1-f\right)\left(w_{11}+w_{22}-2w_{12}\right)x\right.\\ &\quad \left. +f\left(w_{11}-w_{12}\right)+w_{12}-w_{22}\right], \end{aligned} $$

where *x* ^′^ is the frequency of *A* in the next generation, and the mean fitness is given by

1 $$ \begin{aligned} w &= w_{11}x^{2}+2w_{12}x(1-x)+w_{22}(1-x)^{2}\\ &+fx(1-x)\left(w_{11}+w_{22}-2w_{12}\right). \end{aligned}  $$

For the difference *Δ* *w* between the mean fitnesses of two successive generations, we have shown in [14] that

2 $$ \begin{aligned} \Delta w=\frac{x(1-x) J^{2}}{\bar{w}^{2}}\left[w_{11}x+w_{22}(1-x) +\bar{w}\left(\!1- \frac{f\left(w_{11}-w_{22}\right)}{J}\right)\right], \end{aligned}  $$

where *Δ* *w*=*w* ^′^−*w,w* ^′^ is the mean fitness of the next generation and

$$J=(1-f)(w_{11}+w_{22}-2w_{12})x+f(w_{11}-w_{12})+w_{12}-w_{22}. $$

Equation (2) suggests that a sufficient condition for *Δ* *w*>0 is

$$\left(1- \frac{f\left(w_{11}-w_{22}\right)}{J}\right)\ge 0. $$

Thus, for random mating (*f*=0), the mean fitness increases monotonically. Similar observation can be found for the case *w*_11_=*w*_22_ even if *f*≠1. For example, using a different approach, Ziehe and Roberds [18] has shown that, when caused by symmetric homozygous disadvantage at single locus, inbreeding depression is always less than one-third [16]. Here “symmetric” means *w*_11_=*w*_22_. Thus for the populations considered in [18], the mean fitness increases monotonically.

### Inbreeding depression

Now we discuss the variation of mean fitness due to the inbreeding mating. To be consistent with the published literature, from now on, we always make the three genotypes of a population as (1,1−*hs*,1−*s*) in the sequel. Thus the mean fitness *w*_*o*_ with an initial frequency *x* of a randomly mating population is

$$w_{o}=1-2shxy-sy^{2}, $$

and the mean fitness *w*_*s*_ of *x* for the inbreeding mating with inbreeding coefficient *f* is

$$w_{s}=1-2shxy-sy^{2}+fs(1-2h)xy, $$

where *y*=1−*x*. The inbreeding depression is defined by

3 $$ \delta = 1-\frac{w_{s}}{w_{o}} = \frac{fsx(1-x)(1-2h)}{1-2x(1-x)sh-(1-x)^{2}s}.  $$

Based on this definition the inbreeding coefficient is a function of inbreeding coefficient *f*, frequency *x*, degree of dominance *h* and selection coefficient *s*, namely *δ*=*δ*(*x,f,h,s*). We first have the following theory for the changes of mean fitness.

#### **Proposition 1**

Let the genotypes of an inbreeding population be (1,1−*hs*,1−*s*). Then inbreeding mating reduces the mean fitness for the following population:

1. the population with overdominance genotype, or with a partial dominant genotype. That is, inbreeding depression occurs.
2. the population with under-dominance genotype, or with a partial recessive genotype. In this case, no inbreeding depression occurs.

It is well known that an overdominant population has an internal equilibrium. But it is not true for inbreeding populations. In fact, a population with genotypes (1,1−*hs*,1−*s*) has an internal equilibrium if and only if the equation

$$\left(1-f\right)(2h-1)x+fh+1-h=0 $$

has a solution in (0,1). Thus the following Proposition gives the condition for the non-existence of the internal equilibrium.

#### **Proposition 2**

An inbreeding population with over-dominant genotypes (1,1−*hs*,1−*s*) (*h*<0) has no internal equilibrium if and only if

$$h> -\frac{f}{1-f}. $$

For example, if the degree of dominance satisfies −1<*h*<0, there would not be any internal equilibrium for a population of self-fertilization plants with *f*=0.5.

### Multiplicative interactions for polygenic traits

Genes may interact to or regulate each other in a number of different ways. However, genes normally interact multiplicatively for the fitness-reducing effects of homozygosity for deleterious alleles, namely mutant alleles or alleles at loci with overdominance [19, 20, 24]. Accordingly the relative viability *w* of an individual over all loci is assumed to be the product of the viability value *w*_*A*_ for individual locus *A*, namely *w*=*w*_*A*_ *w*_*B*_ *w*_*C*_⋯. All the loci are assumed to assort independently, namely they are unlinked. It is also assumed that there is no mutation at these loci over the period of inbreeding. In addition, we assume that the selective advantage *s* and initial frequency are the same for all loci. Then each *w*_*A*_ can be written as a function of *h*(≤1/*s*), given by

$$w_{A}=w(h)=1-2shxy-sy^{2}+fxy(2hs-s). $$

Then the overall inbreeding mean fitness is

$$w_{s} =\Pi_{h} \left(1-2shxy-sy^{2}+fxy(2hs-s)\right) $$

and that for randomly mating is given by

$$w_{o}=\Pi_{h} \left(1-2shxy-sy^{2}\right). $$

To proceed an theoretic analysis, we write the values of these mean fitness as

$$\ln w_{s} =\sum_{h} \ln w_{s}(h), \quad \ln w_{o} =\sum_{h} \ln w_{o}(h). $$

## Results

### Increase of mean fitness for Mendelian traits

We have described the methods for measuring the fitness of inbreeding population in the previous section. The following theorem gives the condition for the increase of mean fitness.

#### **Theorem 1**

For an inbreeding population with relative genotype fitness

$$w_{11}=1, \quad w_{12}=1+hs, \quad w_{22}=1+s $$

where 0≤*h,s*≤1, the mean fitness increases monotonically for any inbreeding coefficient 0≤*f*≤1.

#### *Proof*

For the difference between the mean fitness of two successive generations (2), we have

$${{}\begin{aligned} \Delta w& =\frac{x(1-x)J^{2}}{\bar w^{2}}\left({\vphantom{\frac{1}{2}}}(1+s)(1-x)\right.\\ & \quad \left. +x+\bar{w} \left(1-\frac{f}{(1-f)(2h-1)x+fh+1-h}\right)\right). \end{aligned}} $$

To prove *Δ* *w*>0, it suffices to show

4 $$ \frac{f}{\left(1-f\right)(2h-1)x+fh+1-h}\le 1.  $$

If 0.5≤*h*≤1, then the inequality (4) holds since $\frac {f}{fh+1-h}\le 1$. On the other hand, when 0≤*h*≤0.5, using the identity

$$\begin{aligned} (1-f)(2h-1)x+fh+1-h&=(1-f)(1-2h)(1-x)-fh+f+h \\ & \,=\,(1\,-\,1f)(1\,-\,2h)(1\,-\,x)\,+\,f\! +\!h(1\,-\,f)\! \ge \!f, \end{aligned} $$

we can check the inequality (4) still holds. Thus we have showed that *Δ* *w*≥0 for any initial frequency *x* and any value of *h* satisfying 0≤*h*≤1. The proof is completed. □

**Fig. 1:**
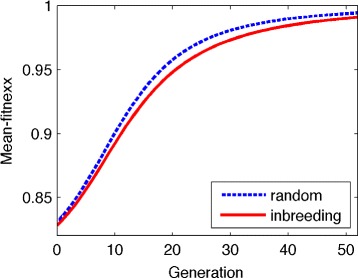
The increasing mean fitness for both random and inbreeding populations with directional selection. *Solid-line*: the mean fitness curve of the inbreeding population. *Dash-line*: the mean fitness curve of the random population. Parameters are selection coefficient *s*=0.2 and the degree of dominant *h*=0.2 (that is, the genotype are (1,0.96,0.8) and inbreeding coefficient *f*=0.25 and initial frequency *x*=0.6

**Fig. 2:**
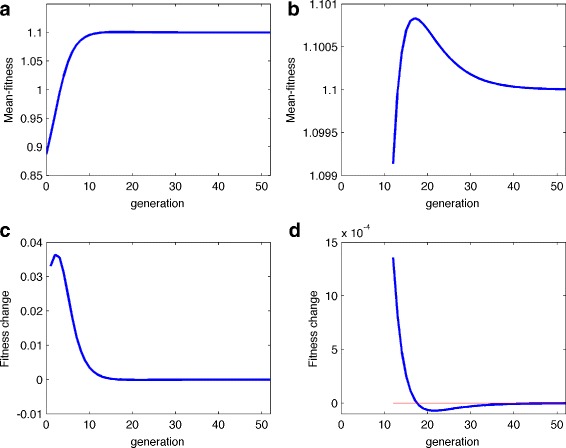
The mean fitness curve for an inbreeding populations with over-dominant genotype. **a** the mean fitness curve of the inbreeding population with selection coefficient *s*=0.2 and the degree of dominant *h*=0.2 (that is, the genotype are (1,1.4,0.8) and inbreeding coefficient *f*=0.25 and initial frequency *x*=0.1). **b** The detailed values in (**a**) from generation 12 to 52. **c** the curve *Δ* *w*(*x*) based on the change of mean-fitness in (**a**). **d** The detailed values in (**c**) from generation 12 to 52

Figure 1 clearly shows that the mean fitness of both randomly mating and inbreeding populations increase monotonically although at each generation, the mean fitness of randomly mating population is higher than that of inbreeding one. This result is consistent with the well-established result for a random-mating population [17]. It also gives the reason for the existence of self-fertilization plants, even though the most extreme form of inbreeding may reduce the heterozygosity of these plants by 50% per generation. Classically, it was known that selfing has two primary advantages over outcrossing [21], namely the reproductive assurance to ensure seed production and transmission advantage by serving as pollen donors for other individuals and for themselves [15]. Since most genotypes of a self-fertilization plant are either partial dominance or partial recessive [8, 22–24], Theorem 1 presents an additional explanation: self-fertilization population increases its mean fitness of population by generating steadily. However, Theorem 1 cannot be extended to the inbreeding population with *h*>1 (overdominance) or *h*<0 (underdominance). The counter examples are given in Table 1, reference [14] or Fig. 2 below.

**Table 1:** Examples for the decreases of mean fitness

f	*x*	*w*_11_	*w*_12_	*w*_22_	$\overline w$	$\overline w'$	*Δ* *w*
0.1	0.62	0.8	1	0.7	0.86802	0.868018	<0
0.25	0.635	0.8	1	0.7	0.85041	0.85040	<0
0.25	0.66	0.8	1	0.7	0.85015	0.85013	<0
0.5	0.7	0.8	1	0.7	0.8225	0.8224	<0
0.5	0.79	0.8	1	0.7	0.82047	0.82045	<0
0.5	0.9	0.7	1	0.4	0.7105	0.7101	<0

On the other hand, the monotonically increasing for mean fitness of a population is not the necessary condition for the population avoiding extinct. Our next goal is to define a quantity which characterize the condition whether a population becomes extinct. To this regard, the mean fitness is denoted as *w*(*x*) to emphasize the importance of the initial frequency *x*. Then we consider the sum of *Δ* *w*(*x*) for all *x*∈(0,1) of an allele *A*, where *Δ* *w*(*x*) is defined by Eq. (2). The *averaged mean fitness* (AMF) of a population is defined by

$$\text{AMF}=\int_{0}^{1} \Delta w(x)dx. $$

The biological meaning of AMF is that *AMF*>0 holds if and only if the population does not become extinct. Thus Theorem 1 in fact shows that AMF for directional inbreeding population is always positive because in this case *Δ* *w*(*x*)>0 for each *x*≠0. The following theorem gives the value of AMP without any additional condition.

#### **Theorem 2**

The AMF of any inbreeding population is positive.

#### *Proof*

The proof is given in Appendix section. □

Figure 2 b and 2 d schematically shows this point, where the genotype fitness is (1,1.4,0.8) and inbreeding coefficient *f*=0.25. From Fig. 2 b, when the population goes to the 8th generation, the frequency *x* becomes 0.6338 and the mean fitness is 1.0831. Then the mean fitness starts to decrease (that is, *Δ* *w*(*x*)<0) until *x* reaches to the equilibrium (*x* ^∗^≈0.6667 in this case). On other hand, Fig. 2 d clearly shows that the positive area which is above the x-axis (and below the curve) is much larger than the negative area which is below the x-axis and above the curve. These figures show that AMF>0 intuitively.

### Inbreeding depression

For inbreeding depression, we give the theorem which provides insights into inbreeding mating.

#### **Theorem 3**

Let (1,1−*hs*,1−*s*) be the genotypes of an inbreeding population with inbreeding coefficient *f*. Then we have

1. If *s*<1, *δ*, as a function of *x* (3), arrives its maximum at $x^{*}= 1- \frac {1-\sqrt {1-s}}{s}=\frac {\sqrt {1-s}}{1+\sqrt {1-s}}$ for $h< \frac {1}{2}$ (partial dominant or over-dominant) and ${\lim }_{x\rightarrow 0}\delta ={\lim }_{x\rightarrow 1}\delta =0$;
2. If *s*=1 and *h*≠1,*δ* is decreasing and ${\lim }_{x\rightarrow 0}\delta =\frac {f(1-2h)}{2(1-h)}$;
3. If *s*=1 and *h*=1, then $\frac {w_{s}}{ w_{o}}$ is not continuous at *x*=0 and ${\lim }_{x\rightarrow 0}\frac {w_{s}}{ w_{o}}= \infty $;
4. If *x*≠0, then *δ*, as a function of *h*, is decreasing and ${\lim }_{h\rightarrow -\infty }\delta = f$;
5. *δ*, as a function of *s*, is strictly increasing;
6. *δ*, as a function of *f*, is strictly increasing.

#### **Remark 1**

This result may provide an explanation to the puzzle for the classical strategy of cultivation. When choosing the offspring (i.e. seeds) of a favourable mutation and then inbreeding between the improved offspring, our results suggest that this inbreeding mating greatly increases the mean fitness of the population. This interesting result explains biological observations but is contradict to the belief that inbreeding mating decreases mean fitness. The key point is that in this case the initial frequency of the favourable mutation normally is very low. However, if the first generation of the population still uses the same selection strategy with the favourable mutation, the initial frequency will be very high, because they all already have the favorable mutation. However, the following Theorem 5 shows that the inbreeding mating for the second generation actually reduces the mean fitness.

### Purging rate

Although selection against highly recessive alleles become more effective due to the rise of selfing rates [25], the amplified selfing rates have little impact on the alleles that are more nearly additive (i.e. *h*=0.5). It was well-known that, for a population with directional selection (i.e., 0≤*h*≤1), inbreeding mating purges deleterious mutants. Here we propose a formula to determine the value of purging rate. For an inbreeding population with genotype (1,1−*hs*,1−*s*) and inbreeding coefficient *f*, the average time *t*(*x,x*_*t*_) required for allele to change the frequency from *x* to *x*_*t*_ is

5 $$ \begin{aligned} t(x,x_{t}) &=\int_{x}^{x_{t}} dx/\Delta x\\ &= \int_{x}^{x_{t} }\frac{1-sy^{2}+xy(2fhs-fs-2hs)}{x(1-x)[(1-f)(2hs-s)x+fhs+s-hs]}dx \\ &= \frac{1}{sa}\left[\frac{1\,-\,s}{b}\ln {\frac{x_{t}}{x}}\,-\,\frac{1}{b\,+\,1}\!\ln\!{\frac{1\,-\,x_{t}}{1\,-\,x}} \,+\,\left[s(K\,+\,2)\,-\,\frac{1\,-\,s}{b}\,+\,\frac{1}{b\,+\,1}\,-\,s\right] \cdot\right.\\&\qquad\qquad\qquad\qquad\qquad\qquad\qquad\qquad\qquad\left.\ln\frac{x_{t}+b}{x+b}\right] \end{aligned}  $$

where $a=\left (1-f\right)(2h-1),\,b=\frac {fh+1-h}{a}, K=2fh-f-2h$ and *f* satisfies $-\frac {f}{1-f}\le h\le 1$. When 0≤*h*≤1, the purging rate satisfies

$${{} {\begin{aligned} t(x,x_{t})\!\le \!t(x,x_{t})|_{f=0}\,=\,\int_{x}^{x_{t} }\frac{1\,-\,sy^{2}\,-\,2xyhs}{x(1\,-\,x)[(2hs\,-\,s)x\,+\,s\,-\,hs]}dx. \end{aligned}}} $$

**Fig. 3:**
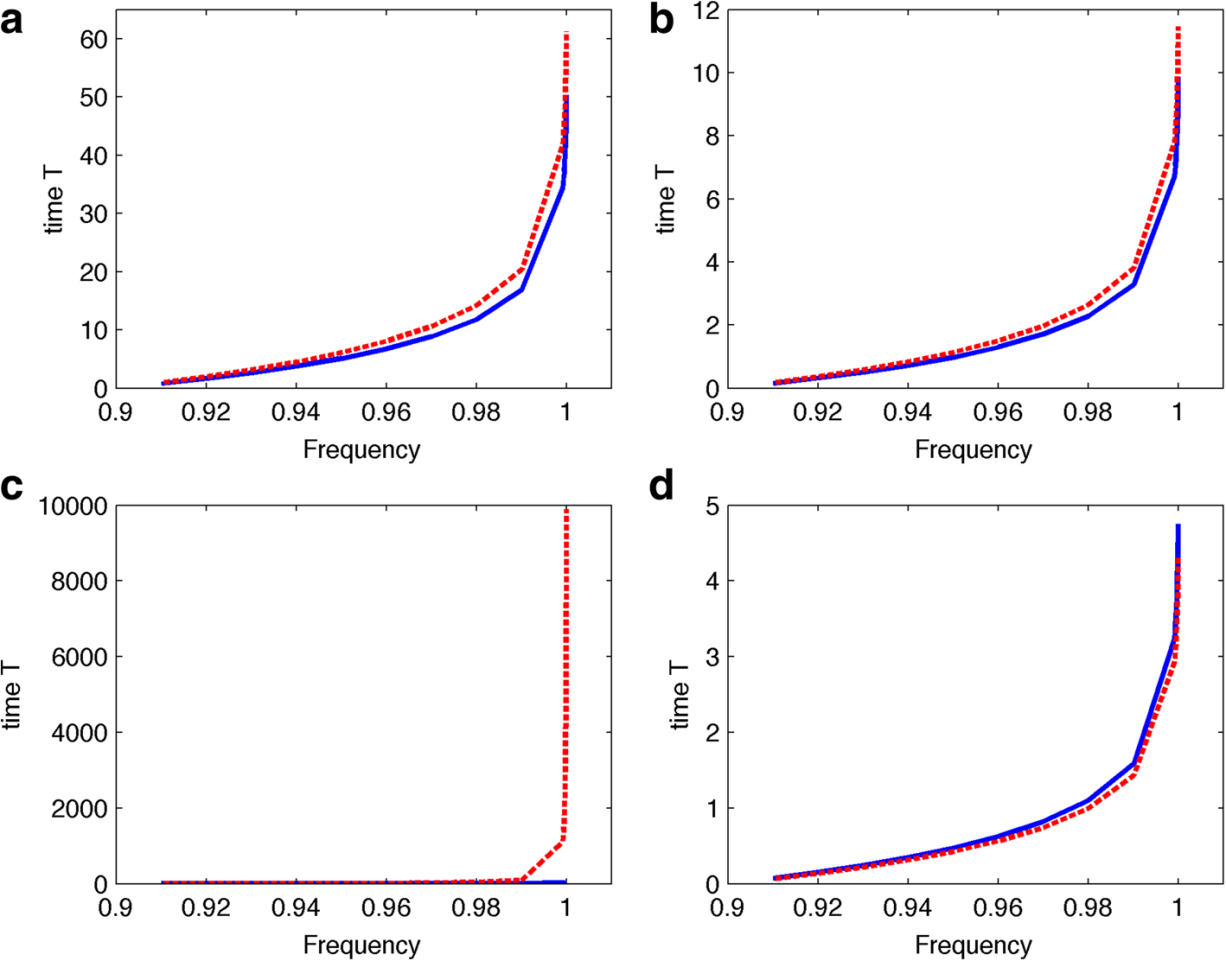
The curve for the time required from initial frequency *x*=0.9 to the given frequency in the figure. **a** Selection coefficient *s*=0.5 and the degree of dominant *h*=0.3 (that is, genotype (1,0.85,0.5)). **b** *s*=1 and *h*=0.3 (genotype (1,0.7,1)). **c** *s*=1 and *h*=0 (genotype (1,1,0)). **d** *s*=1 and *h*=0.8 (genotype (1,0.2,0)). (dash-time: randomly mating population; solid-line: inbreeding population with inbreeding coefficient *f*=0.25)

Figure 3 gives the calculated time *t*(*x,x*_*t*_) based on different genotypes and different frequency *x*_*t*_. Numerical results show that the random mating population need more time than the inbreeding population from a fixed initial frequency *x*=0.9 to a new frequency, which suggests that inbreeding mating purges deleterious mutants more effectively than randomly mating. In particular, Fig. 3(c) suggests that the purging strength of the inbreeding population is substantial higher than that of the random mating population. In addition, Comparing the three cases with *h*=0,0.3,0.8, Fig. 3 suggests that a small value of *h* leads to a large purging rate. These results may provide a reason for the existence of so many selfing fertilisation plants, though these plants have various degree of inbreeding depression. This may be one of the advantages of selfing fertilisation plants.

### Multiplicative interactions for polygenic traits

We first give an example to show that the theory established in the previous section may not be able to explain some experimental observations. For example, for an selfing population with inbreeding coefficient *f*=0.5, nature selection pressure *s*=0.05 and *h*=0 (i.e. a completely dominant mutant), the inbreeding depression at a single locus (3) is *δ*=0.006. However, the observation in natural populations shows that a completely recessive mutant usually will cost around 20−30*%* inbreeding depression [9, 23, 24]. This difference suggests that the trait may be determined by multi-genes.

In our previous study, we proposed a theorem for the inbreeding mating without proof. The following result is an updated version of Theorem 3.1 in [14].

#### *Proof*

For each partial dominance genotype (1,1−*hs*,1−*s*) with 0≤*h*<0.5, there is a partial recessive genotype (1,1−(1−*h*)*s*,1−*s*). It suffices to show

$${} \left(\ln w_{s}(h) - \ln w_{o}(h)\right)+ \left(\ln w_{s}({1-h}) - \ln w_{o}({1-h})\right)\ge 0. $$

Let *A*=*w*_*o*_(0.5)=1−*sxy*−*sy* ^2^=1−*sy,a*=*sxy*(1−2*h*) and *z*=*fsxy*(1−2*h*)=*fa*. Then we have that

$$\begin{array}{@{}rcl@{}} \ln w_{s}(h)&=&\ln(A+a-z),\\ \ln w_{o}(h)&=&\ln(A+a),\\ \ln w_{s}(1-h)&=&\ln(A-a+z),\\ \ln w_{o}(1-h)&=&\ln(A-a). \end{array} $$

In addition, the log-ratio of mean fitness is

$$\begin{aligned} &(\ln w_{s}(h) - \ln w_{o}(h))+ (\ln w_{s}({1-h}) - \ln w_{o}({1-h}))\\ &\qquad\qquad\qquad\qquad\qquad\quad=\ln\left(1-\frac{z}{A+a}\right)+\ln\left(1+\frac{z}{A-a}\right) \\ &\qquad\qquad\qquad\qquad\qquad\quad=\ln\left(1\,+\,\frac{z}{A-a}-\frac{z}{A+a}-\frac{z^{2}}{A^{2}-a^{2}}\right)\\ &\qquad\qquad\qquad\qquad\qquad\quad=\ln\left(1+\frac{fa^{2}(2-f)}{A^{2}-a^{2}}\right)\ge 0 \end{aligned} $$

since *A* ^2^≥*a* ^2^. Hence the proof is completed.

#### **Theorem 4**

Assume that a polygenic trait is determined by the genotypes whose relative fitness is (1,1−*hs*,1−*s*), where the selective advantage coefficient *s* is fixed and the degree of dominance *h* is uniformly distributed over [0,1] (i.e., the trait is determined by the dominant-recessive genotypes). Then inbreeding mating does not decrease the mean fitness of population. □

Note that the inbreeding depression *δ* is an allele-specific property. However Theorem 4 shows a genotype-specific property which is independent of the choice of alleles.

From Proposition 1, we immediately have the following results.

#### **Proposition 3**

If a polygenic trait is determined by partial dominant or over-dominant genotypes, inbreeding mating reduces the mean fitness of the population.

#### **Theorem 5**

Assume that a polygenic trait is determined by the genotypes whose relative fitnesses are (1,1−*hs*,1−*s*), where the selective advantage coefficient *s* is fixed and the degree of the dominance *h* is uniformly distributed over [ −1,1]. Then inbreeding mating reduces the mean fitness of the population for all initial frequency *x* satisfying

6 $$ x\ge \frac{4-3f}{6-3f}.  $$

#### *Proof*

The proof is given in Appendix section. □

#### **Remark 2**

The restriction (6) in Theorem 5 is a sufficient condition, but it can not be omitted. For example, suppose *s*=1,*f*=0.5 and a polygenic trait is determined by (1,1−*hs*,1−*s*) with *h*=−1,−0.8,−0.6,…,1. When *x*=0.1, we have

$$\begin{aligned} \ln(w_{s})&=\ln(\Pi_{-5}^{5} w_{s}(0.2n))=-22.23< -21.98\\ &=\ln(\Pi_{-5}^{5} w_{o}(0.2n))=\ln(w_{o}), \end{aligned} $$

which suggests that inbreeding mating reduces the fitness. Note that $\frac {4-3f}{6-3f}\approx 0.56$. This means that the above condition (6) is not necessary. However, when *x*=0.05, we have ln(*w*_*s*_)=−29.77>−30.15= ln(*w*_*o*_) and hence *w*_*s*_>*w*_*o*_. This means that the condition (6) cannot be removed.

Recent studies have showed that there are many overdominant loci in rice [3, 4, 26]. In addition, overdominance and epistasis might play an important role as the genetic basis of heterosis in Brassica rapa [27]. The following theorem investigates the relationship between the number of genotypes and the mean fitness.

#### **Theorem 6**

If the set of genotypes which determine a polygenic trait contains enough over-dominant genotypes, inbreeding mating reduces the mean fitness of the population for any given initial frequency *x*≠0, that is, *w*_*s*_≤*w*_*o*_.

#### *Proof*

The proof is given in Appendix section. □

However, in natural populations, there may not be many overdominance genotypes [28]. Another restriction for over dominance (1,1−*hs*,1−*s*) is that *h* may not be very small. A well-known example is Sickle cell anaemia which occurs when a particular pair of genes carry the ’sickle-cell trait’ which has not been eliminated from the human population by selection. The reason is that there is only one pair of genes carrying the sickle-cell trait and individuals (“carrier”) is highly resistant to malaria. On the other hand, a person whose genes do not carry the sickle-cell trait is susceptible to malaria. For example, according to the data from the World Health Organization, the carrier frequency ranges from 10 to 40% across equatorial Africa. The genotype’s fitness is 1,1−*h* and 0 with −1.35≤*h*≤−1.05. Thus *h* (≥−2*s,s*=1) is not very small.

## Discussions

We now discuss other factors for determining inbreeding depression. Suppose that initially we have two homozygous, inbred lines *P*_1_ and *P*_2_ which are used as parents. Let $\bar P_{1}>\bar {P}_{2}$, where $\bar {P}$ is the mean fitness or fitness-related trait of *P*. The genotypes of *P*_1_ and *P*_2_ for this gene are *A* ^+^ *A* ^+^ and *A* ^−^ *A* ^−^ while their hybrid is *A* ^+^ *A* ^−^. Let the average phenotype of the two parents be *m* and the additive and dominance genetic component of means (the averaged phenotypic value) be $a_{A} \left (=\bar {P}_{1}-m\right)$ and $d_{A}\left (=\bar {F}_{1}-m\right)$, respectively, where *F*_1_ is the first generation of hybrid offsprings of *P*_1_ and *P*_2_ and $\bar F_{1}$ is the mean of the trait of *F*_1_. Suppose *d*_*A*_>*a*_*A*_>0, that is, *P*_1_ is overdominant to *P*_2_. If inbreeding mating, the mean value is $\left (\bar {P}_{1}+\bar {P}_{2}\right)/2=m$. However, for cross mating, the mean value is $\bar {F}_{1}$ which is greater than *m*. Consider a natural population consisting of equal number of *P*_1_ and *P*_2_. Then inbreeding reduces the mean value of fitness or fitness-related, namely this population exhibits inbreeding depression. On the other hand, Theorem 4 shows that a natural population with few over-dominant genotypes does not exhibit severe inbreeding depression. The next result provides a reason to explain this difference.

### **Theorem 7**

If a natural inbreeding population does not show a high level of inbreeding depression (i.e., *δ*≤0.2), then most fitness or fitness related loci (QTL) of it exhibits additive and dominant-recessive genotypes.

We conclude that inbreeding depression is determined by not only the inbreeding coefficient but also the genetical structure of the population. This result is consistent with the observation that, in many mixed-mating plant populations, selfing (≈0.2), outcrossing (≈0.4) and the interm1diate (≈0.4) reaches at an equilibrium [29].

Let us consider an example. Goodwillie and Knight [2] measured inbreeding depression in three populations, namely Lake Hennessey (LH), Wantrup Researve (WR) and Ida Clayton Road (IC) of Leptosiphon, whose mean outcrossing rate are 0.06,0.37 and 0.69, respectively. Significant inbreeding depression was observed for the proportion of fertilized ovules that developed into seeds only occurred in the more outcrossing WR and IC populations. Theorem 7 predicts that most genes in population LH are additive and dominant-recessive and both LH and WR have many over-dominant genes.

Another novel contribution of this work is the study for inbreeing depression with polygenic traits. Our theoretical results have shown that inbreeding depression also depends on the structure of the genotypes. The theoretical results support the belief that inbreeding depression is caused more like by over-dominant mechanism rather than dominance-recessive mechanism. An application of these results is to explain why Caenorhabditis remanei has demonstrated to suffer severely from inbreeding depression, while its hermaphroditic relative C. elegans has not [22, 30]. In addition, Theorem 5 shows that inbreeding depression is also determined by the initial frequency, which, for cultivation of improved varieties with a favorable mutation, explains why inbreeding mating does not reduce the mean fitness of the first generation, but does reduce the mean fitness of the second generation. These results may have potential application for cultivation of improved varieties.

## Conclusions

In this work we developed mathematical approaches to investigate the the variations of inbreeding population fitness under various conditions. For inbreeding population with a single locus, we proved that the mean fitness increases monotonically for directional selection, which extends the existing result for the random mating substantially [17]. Our results showed that, if most genotypes are additive, inbreeding mating (selfing) does not produce inbreeding depression. In addition, we defined the averaged mean fitness (AMF). Using this concept, we have successfully shown monotonical increase of the averaged mean fitness for any inbreeding population. These results can be applied to study the Mendelian trait or unlinked polygenic trait, and provide the reason to explain why inbreeding mating does not have serious impact on the mean fitness of population. For inbreeding depression with single trait, we have presented an explicit formula to calculate purging rate. Computing results suggest that inbreeding mating does purge deleterious mutants more efficiently than randomly mating does.

## Appendix: Theoretic Proofs

### **Theorem 2**

The averaged mean fitness (AMF) of any inbreeding population is positive, that is,

$${AMF} =\int_{0}^{1}\Delta w(x) > 0. $$

### *Proof*

The proof for *AMF*>0 is equivalent to prove

$$\begin{aligned} &\int_{0}^{1}x(1-x) J^{2}\left[w_{11}x+w_{22}(1-x)+ w\left(1-\frac{f(w_{11}-w_{22})}{J}\right)\right]dx\\ &= \int_{0}^{1}x(1\,-\,x) J\left[(w_{11}x\,+\,w_{22}(1-x))J \,+\, w(J- f(w_{11}-w_{22}))\right]dx>0 \end{aligned} $$

since min{*w*(*x*)|*x*∈[0,1]}>0. We first define

$$\begin{aligned} S(f)&= \int_{0}^{1}x(1-x) J\left[(w_{11}x+w_{22}(1-x))J +\right.\\&\qquad\qquad\qquad\qquad\qquad \left.w(J- f(w_{11}-w_{22}))\right]dx \end{aligned} $$

and let *w*_11_=1−*s,w*_12_=1 and *w*_22_=1−*t*, where 0≤*s,t*≤1. Then we have that

$$\begin{aligned} S(f)&=\frac{1}{420}\left[\left(\,-\,24\,-\,12f\,-\,2f^{2}\,+\,3f^{3}\right)\left(t^{3}\,+\,s^{3}\right)+\left(14+21f+7f^{2}\right)\left(t^{2}+s^{2}\right)+\right.\\ & \quad \left.+\left(12+34f-6f^{2}-5f^{3}\right)\left(t^{2}s+ts^{2}\right)+28(t-s)^{2}+\left(14f^{2}-98f\right)ts\right]\\ &=\frac{1}{420}\left[\left(-24-12f-2f^{2}+3f^{3}\right)\left(t^{3}+s^{3}\right)\right.\\&\quad\left.+\left(12+34f-6f^{2}-5f^{3}\right)\left(t^{2}s+ts^{2}\right)+\right.\\ &\quad \left.+\left(14-28f+14f^{2}\right)\left(t^{2}+s^{2}\right)+\left(28+49f-7f^{2}\right)\left(t-s\right)^{2}\right]\\ &\ge\frac{1}{420}\left[{\vphantom{\frac{1}{2}}}14\left(1-f\right)^{2}\left(t^{2}+s^{2}-t^{3}-s^{3}\right)+\right.\\ &\quad \left.+\left(10\,+\,40f\,-\,12f^{2}\,-\,3f^{3}\right)\left(t\,-\,s\right)^{2}\cdot \left(\!\frac{28\,+\,49f\,-\,7f^{2}}{10\,+\,40f\,-\,12f^{2}\,-\,3f^{3}}-(t+s)\right)\! \right]. \end{aligned} $$

□

We consider two cases. (1) Let *w*_11_+*w*_22_≥0.111 (equivalently, *t*+*s*≤1.889). In this case, note that (10+40*f*−12*f* ^2^−3*f* ^3^)>0 and

$$\frac{28+49f-7f^{2}}{10+40f-12f^{2}-3f^{3}}>1.889 $$

hold for 0≤*f*≤1. Thus *S*(*f*)>0 hold.

(2) Let *w*_11_+*w*_22_≤0.111. Then we have that

$$\begin{aligned} S(f)&\ge 14(1-f)^{2}\left(t^{2}+s^{2}-t^{3}-s^{3}\right)\\&\quad+\left(10+40f-12f^{2}-3f^{3}\right)(t-s)^{2}(1.889-2) \end{aligned} $$

and 0.889≤*s,t*≤1 holds.

In addition, let *t*≥*s* and write

$$\begin{aligned} P(f)&=14(1-f)^{2}(t^{2}+s^{2}-t^{3}-s^{3})\\ & \quad+(10+40f-12f^{2}-3f^{3})(t-s)^{2}(1.889-2). \end{aligned} $$

Then *P*(*f*) is a decreasing function of *t* since

$$\begin{aligned} &P'(f)_{t} = 14(1-f)^{2}(2t-3t^{2})\\&+(10+40f-12f^{2}-3f^{3})2(t-s)(1.889-2)\le 0. \end{aligned} $$

Hence we have, for *f*≤0.8,

$$\begin{aligned} S(f)\ge P(f)&\ge P(f)_{t=1}= 14(1-f)^{2}(s^{2}-s^{3})\\&-0.111(10+40f-12f^{2}-3f^{3})(1-s)^{2}. \end{aligned} $$

Also note that *P*(*f*)_*t*=1_ is a decreasing function of *f* and [*P*(*f*)_*t*=1_]_*f*=0.8_>0 for *s*≥0.89.

Thus we further have

$${} {{\begin{aligned} &S(f)\ge P(f)\ge P(f)_{t=1}\ge [P(f)_{t=1}]_{f=0.8}\\ &=0.56(s^{2}\,-\,s^{3})\,-\,0.111(10\,+\,40*0.8\,-\,12\!*\!0.8^{2}\,-\,3*0.8^{3})\\&\qquad(1-s)^{2}\ge 0. \end{aligned}}} $$

For *f*≥0.8, we have

$$\frac{28+49f-7f^{2}}{10+40f-12f^{2}-3f^{3}}>1.913 $$

and note that the left hand side function of above equation is increasing for *f*≥0.8 and

$$\begin{aligned} S(f)&\ge 14(1-f)^{2}(s^{2}-s^{3})-0.087*\\&(10+40f-12f^{2}-3f^{3})(1-s)^{2}\ge 0 \end{aligned} $$

if *f*≤0.85. However, for *f*≥0.85, we have

$$\frac{28+49f-7f^{2}}{10+40f-12f^{2}-3f^{3}}>1.928 $$

and

$$\begin{aligned} S(f)&\ge 14(1-f)^{2}(s^{2}-s^{3})-0.072*\\&(10+40f-12f^{2}-3f^{3})(1-s)^{2}\ge 0. \end{aligned} $$

By repeating this procedure, we can prove *S*(*f*)>0 for *f*<1. Finally, if *w*_11_≠*w*_22_, it is easy to check that

$${{} {\begin{aligned} S(1)\,=\, 35(t\,-\,s)^{2}(2\,-\,t\,-\,s)\,=\,35(w_{11}\,-\,w_{22})^{2}(w_{11}\,+\,w_{22})>0 \end{aligned}}} $$

Note that the unique equilibrium

$$x^{*}=\frac{t-fs}{(1-f)(s+t)} $$

and the mean fitness reaches its maximum value at

$$\bar x= \frac{2t-fs-ft}{2\left(1-f\right)\left(s+t\right)}. $$

In addition, *Δ* *w*(*x*)<0 holds if and only if $x\in (\bar x, x^{*})$ with

$$\bar x- x^{*} =\frac{f(s-t)}{2\left(1-f\right)\left(s+t\right)}. $$

A similar argument can be applied to the case of under-dominance populations. Thus the proof of Theorem 2 is completed.

### **Theorem 3**

Assume that a polygenic trait is determined by the genotypes whose relative fitnesses are (1,1−*hs*,1−*s*), where the selective advantage coefficient *s* is fixed and the degree of the dominance *h* is uniformly distributed over [−1,1]. Then inbreeding mating reduces the mean fitness of the population for all initial frequency *x* satisfying

$$x\ge \frac{4-3f}{6-3f}. $$

### *Proof*

By the assumption, for each 0≤*h*≤1, there is an over-dominant genotype (1,1+*hs*,1−*s*). It suffices to show

$$ \left(w_{s}(h) w_{s}(-h)\right)- \left(w_{o}({h}) w_{o}({-h})\right)\le 0 $$

for any 0≤*h*≤1. In addition, we also have

$$\begin{array}{@{}rcl@{}} w_{s}(h)w_{s}(-h)&=&\left(1-sy^{2}-fsxy\right)^{2}-4s^{2}x^{2}y^{2}(f-1)^{2}h^{2}\\ w_{o}(h)w_{o}(-h)&=&\left(1-sy^{2}\right)^{2}-4s^{2}x^{2}y^{2}h^{2}. \end{array} $$

Thus we further have

$$\begin{aligned} (w_{s}(h) w_{s}(-h))- (w_{o}({-h}) w_{o}({-h})) &=\left(1-sy^{2}-fsxy\right)^{2}-\left(1-sy^{2}\right)^{2} \\&\quad- 4s^{2}x^{2}y^{2}h^{2}\left((f-1)^{2}-1\right) \\ &= -fsxy\left(2\,-\,2sy^{2}\,-\,fsxy\right)\,-\,4s^{2}x^{2}y^{2}h^{2}(f\,-\,2)~f. \end{aligned} $$

Hence it suffices to show either

$$\left(2-2sy^{2}-fsxy\right)+4sxyh^{2}\left(f-2\right)\ge 0, $$

or

$$\left(2-2sy^{2}-fsxy\right)+4sxy\left(f-2\right)\ge 0. $$

Note that the left hand side of the last inequality is

$$\begin{array}{@{}rcl@{}} LHS &=&\left(fs-4sf+8s-2s\right)y^{2}-\left(fs-4sf+8s\right)y+2\\ &\ge &\left(f-4f+8-2\right)y^{2}-\left(f-4f+8\right)y+2\\ &=&\left(6-3f\right)y^{2}-\left(8-3f\right)y+2, \end{array} $$

since it is a decreasing function of *s*.

Let $\bar y$ be the smaller root of (6−3*f*)*y* ^2^−(8−3*f*)*y*+2. Then we have $\bar y=\frac {2}{6-3f}$ and (6−3*f*)*y* ^2^−(8−3*fh* ^2^)*y*+2 is a decreasing function for $y\le \bar y$.

We further have that

$${} \left(\!6-3f\right)\!y^{2}-\left(8-3f\right)y+2 \ge\! \left(6-3f\right)\bar y^{2}-\left(8-3f\right)\bar y+2 \ge 0 $$

for all $y\le \frac {2}{6-3f}$. Thus

$$\left(w_{s}(h) w_{s}(-h)\right)- \left(w_{o}({h}) w_{o}({-h})\right)\le 0 $$

holds and hence *w*_*s*_≤*w*_*o*_ holds for $y\le \frac {2}{6-3f}$ or equivalently for $x\ge \frac {4-3f}{6-3f}$. The proof is completed. □

### **Theorem 4**

If the set of genotypes which determine a polygenic trait contains enough over-dominant genotypes, then inbreeding mating reduces the mean fitness of the population for any given initial frequency *x*≠0, that is, *w*_*s*_≤*w*_*o*_.

### *Proof*

It suffices to show ln*w*_*s*_− ln*w*_*o*_≤0. In fact, we first have

$$\begin{aligned} \ln w_{s} -\ln w_{o} &=\sum\limits_{h}(\ln w_{s}(h)-\ln w_{o}(h))\\ &=\sum\limits_{0}^{1}(\ln w_{s}(h)-\ln w_{o}(h))+\sum\limits_{-N}^{0}(\ln w_{s}(h)-\ln w_{o}(h)). \end{aligned} $$

Note that if *h*_1_≤*h*_2_≤0, then

$$\left(\ln w_{s}\left(h_{1}\right)-\ln w_{o}\left(h_{1}\right)\right)\le \left(\ln w_{s}\left(h_{2}\right)-\ln w_{o}\left(h_{2}\right)\right). $$

In fact, we have that

$$\begin{aligned} (w_{s}(h_{1})- w_{o}(h_{1})) &= \left(1\,-\,sy^{2}\,-\,fsxy\,+\,2sxy(f-1)h_{1}\right)-\left(1-sy^{2}-2sxyh_{1}\right)\\ &=fsxy(2h_{1}-1)\\ &\le fsxy(2h_{2}-1)=w_{s}(h_{2})-w_{o}(h_{2}). \end{aligned} $$

Thus we have that

$$\begin{aligned} \ln w_{s} -\ln w_{o} & = \sum\limits_{h=0}^{1}\left(\ln w_{s}(h)\,-\,\ln w_{o}(h)\right)\,+\,\sum\limits_{h=-N}^{0}\left(\ln w_{s}(h)-\ln w_{o}(h)\right)\\ &\le \sum\limits_{0}^{1}(\ln w_{s}(h)-\ln w_{o}(h))+ N(\ln w_{s}(0)-\ln w_{o}(0)). \end{aligned} $$

Since (ln*w*_*s*_(0)− ln*w*_*o*_(0))<0 for *x*≠0,1 by Proposition 1(2), we have that

$$\sum_{0}^{1}\left(\ln w_{s}(h) -\ln w_{o}(h)\right)+ N\left(\ln w_{s}(0)-\ln w_{o}(0)\right)\le 0 $$

for enough large *N*. Thus ln*w*_*s*_− ln*w*_*o*_≤0 holds, that is, ln*w*_*s*_≤ ln*w*_*o*_ holds. The proof is completed. □
